# Supplementary material for: Heat adaptation of phage T7 under an extended genetic code
Source: Virus Evol. 2021 Dec 1;7(2):veab100. doi: 10.1093/ve/veab100 (PMC8923235; doi:10.1093/ve/veab100)
Supplement: veab100_Supp [file veab100_supp.zip › Sequencing_Analysis.zip!/Sequencing_Analysis/A1/marginal.html]

BRESEQ :: Marginal Predictions


|  |  |
| --- | --- |
|  | ***breseq***  version 0.30.1    mutation predictions \| marginal predictions \| summary statistics \| genome diff \| command line log |

| Marginal read alignment evidence | | | | | | | | | | | |
| --- | --- | --- | --- | --- | --- | --- | --- | --- | --- | --- | --- |
|  | seq id | position | | ref | new | freq | score (cons/poly) | reads | annotation | genes | product |
| \* | NC\_001604.1 | 24,346 | 0 | C | T | 77.9% | 4251.2 / inf | 2870 | intergenic (–/–) | *–/–* | –/– |
| \* | NC\_001604.1 | 27,932 | 0 | T | C | 67.8% | 4186.3 / inf | 3427 | intergenic (–/–) | *–/–* | –/– |
| \* | NC\_001604.1 | 27,111 | 0 | T | C | 67.6% | 3289.3 / inf | 2789 | intergenic (–/–) | *–/–* | –/– |
| \* | NC\_001604.1 | 26,148 | 0 | A | G | 62.8% | 2456.9 / inf | 2705 | intergenic (–/–) | *–/–* | –/– |
| \* | NC\_001604.1 | 29,697 | 0 | C | T | 61.0% | 755.1 / inf | 2651 | intergenic (–/–) | *–/–* | –/– |
| \* | NC\_001604.1 | 28,493 | 0 | A | G | 44.5% | 449.7 / inf | 2782 | intergenic (–/–) | *–/–* | –/– |
| \* | NC\_001604.1 | 33,022 | 0 | G | T | 43.2% | 3195.7 / inf | 3683 | intergenic (–/–) | *–/–* | –/– |
| \* | NC\_001604.1 | 35,254 | 0 | A | G | 36.9% | 1733.4 / inf | 2801 | intergenic (–/–) | *–/–* | –/– |
| \* | NC\_001604.1 | 33,069 | 0 | G | T | 26.2% | 7113.9 / inf | 3808 | intergenic (–/–) | *–/–* | –/– |
| \* | NC\_001604.1 | 35,515 | 0 | A | C | 22.7% | 2999.6 / inf | 2941 | intergenic (–/–) | *–/–* | –/– |
| \* | NC\_001604.1 | 28,943 | 0 | C | A | 21.4% | 5538.9 / inf | 2669 | intergenic (–/–) | *–/–* | –/– |
| \* | NC\_001604.1 | 24,386 | 0 | G | T | 21.1% | 5664.8 / inf | 2802 | intergenic (–/–) | *–/–* | –/– |
| \* | NC\_001604.1 | 20,976 | 0 | A | C | 20.8% | 2835.8 / inf | 2566 | intergenic (–/–) | *–/–* | –/– |
| \* | NC\_001604.1 | 13,582 | 0 | T | A | 20.7% | 3838.1 / inf | 2343 | intergenic (–/–) | *–/–* | –/– |
| \* | NC\_001604.1 | 13,579 | 0 | C | T | 20.5% | 4406.9 / inf | 2320 | intergenic (–/–) | *–/–* | –/– |

| Marginal new junction evidence (lowest skew 10 of 13 shown) | | | | | | | | | | | |
| --- | --- | --- | --- | --- | --- | --- | --- | --- | --- | --- | --- |
|  | | seq id | position | reads (cov) | reads (cov) | score | skew | freq | annotation | gene | product |
| \* | ? | NC\_001604.1 | = 13455 | 684 (0.220) | 103 (0.030) | 80/586 | 17.8 | 20.7% | intergenic (–/–) | *–/–* | –/– |
|  | ? | NC\_001604.1 | 14331 = | 121 (0.040) |  |  |  |  | intergenic (–/–) | *–/–* | –/– |
| \* | ? | NC\_001604.1 | = 27753 | 2681 (0.920) | 176 (0.060) +CGCTATATCTGG | 45/544 | 24.3 | 6.0% | intergenic (–/–) | *–/–* | –/– |
|  | ? | NC\_001604.1 | 27754 = | 2915 (0.940) |  |  |  |  | intergenic (–/–) | *–/–* | –/– |
| \* | ? | NC\_001604.1 | = 13244 | 2141 (0.690) | 49 (0.020) | 39/582 | 27.3 | 4.5% | intergenic (–/–) | *–/–* | –/– |
|  | ? | NC\_001604.1 | 14067 = | 6 (0.000) |  |  |  |  | intergenic (–/–) | *–/–* | –/– |
| \* | ? | NC\_001604.1 | 295 = | 3899 (1.260) | 25 (0.010) | 15/560 | 36.7 | 0.68% | intergenic (–/–) | *–/–* | –/– |
|  | ? | NC\_001604.1 | = 281 | 3655 (1.270) |  |  |  |  | intergenic (–/–) | *–/–* | –/– |
| \* | ? | NC\_001604.1 | = 36882 | 3450 (1.120) | 16 (0.010) | 15/582 | 37.4 | 0.48% | intergenic (–/–) | *–/–* | –/– |
|  | ? | NC\_001604.1 | 37265 = | 3289 (1.100) |  |  |  |  | intergenic (–/–) | *–/–* | –/– |
| \* | ? | NC\_001604.1 | = 10002 | 2433 (0.790) | 15 (0.000) | 14/594 | 38.4 | 0.51% | intergenic (–/–) | *–/–* | –/– |
|  | ? | NC\_001604.1 | 10186 = | 3422 (1.120) |  |  |  |  | intergenic (–/–) | *–/–* | –/– |
| \* | ? | NC\_001604.1 | = 13578 | 23 (0.010) | 22 (0.010) | 13/584 | 38.7 | 15.8% | intergenic (–/–) | *–/–* | –/– |
|  | ? | NC\_001604.1 | 14348 = | 212 (0.070) |  |  |  |  | intergenic (–/–) | *–/–* | –/– |
| \* | ? | NC\_001604.1 | = 16604 | 2883 (0.930) | 10 (0.000) | 9/590 | 41.6 | 0.35% | intergenic (–/–) | *–/–* | –/– |
|  | ? | NC\_001604.1 | 17028 = | 2874 (0.950) |  |  |  |  | intergenic (–/–) | *–/–* | –/– |
| \* | ? | NC\_001604.1 | = 9789 | 3293 (1.200) | 306 (0.110) +24 bp | 4/486 | 42.4 | 8.3% | intergenic (–/–) | *–/–* | –/– |
|  | ? | NC\_001604.1 | 9790 = | 3613 (1.170) |  |  |  |  | intergenic (–/–) | *–/–* | –/– |
| \* | ? | NC\_001604.1 | = 13568 | 84 (0.030) | 12 (0.000) | 7/586 | 43.0 | 8.7% | intergenic (–/–) | *–/–* | –/– |
|  | ? | NC\_001604.1 | 14339 = | 169 (0.060) |  |  |  |  | intergenic (–/–) | *–/–* | –/– |
